# Common Failures in Hydraulic Kaplan Turbine Blades and Practical Solutions

**DOI:** 10.3390/ma16093303

**Published:** 2023-04-23

**Authors:** Waleed Khalid Mohammed Ridha, Kazem Reza Kashyzadeh, Siamak Ghorbani

**Affiliations:** 1 Department of Mechanical Engineering Technologies, Academy of Engineering, Peoples’ Friendship University of Russia (RUDN University), 6 Miklukho-Maklaya Street, 117198 Moscow, Russia; 2 Department of Transport, Academy of Engineering, Peoples’ Friendship University of Russia (RUDN University), 6 Miklukho-Maklaya Street, 117198 Moscow, Russia

**Keywords:** hydropower, Kaplan hydro turbine, turbine blade, internal object damage, foreign object damage, nanostructured coatings

## Abstract

Kaplan turbines, as one of the well-known hydraulic turbines, are generally utilized worldwide for low-head and high-flow conditions. Any failure in each of the turbine components can result in long-term downtime and high repair costs. In a particular case, if other parts are damaged due to the impact of the broken blades (e.g., the main shaft of the turbine), the whole power plant may be shut down. On the other hand, further research on the primary causes of failures in turbines can help improve the present failure evaluation methodologies in power plants. Hence, the main objective of this paper is to present the major causes of Kaplan turbine failures to prevent excessive damage to the equipment and provide practical solutions for them. In general, turbines are mainly subjected to both Internal Object Damage (IOD) and Foreign Object Damage (FOD). Accordingly, this paper presents a state-of-the-art review of Kaplan turbine failures related to material and physical defects, deficiencies in design, deficits in manufacturing and assembly processes, corrosion failures, fatigue failure, cavitation wear, types of cavitation in hydro turbines, hydro-abrasive problems, and hydro-erosion problems. Eventually, the authors have attempted to discuss practical hints (e.g., nanostructured coatings) to prevent damages and improve the performance of Kaplan turbines.

## 1. Introduction

Turbine is the most essential mechanical component of any hydropower generating unit. This device converts the kinetic energy of running water into the rotary motion required to turn a generator, and then generates electricity. According to the published reports, cavitation [1,2,3], silt erosion [4,5,6,7], and fatigue [8,9,10,11] are the common reasons for damages to hydro turbines, specifically Kaplan turbines. Since the finding of the first type of hydro turbine (Francis) in 1848 [12], the erosion of hydraulic machines due to cavitation and solid particles has been challenging for engineers involved in the design, construction, operation, and maintenance of HPPs. Moreover, cavitations, including tip clearance cavitation [13] and tip vortex cavitation [14], might take place in hydro turbines due to the presence of hard particles such as feldspar and quartz. This is associated with the fact that the pressure inside the hydro unit frequently drops low enough to equal the vapor pressure of water [15]. In addition, the bubbles generated in the region of gas- or vapor-filled cavities affect the flow stability, resulting in vibration of the hydro unit [16]. Other consequences of the cavities may be noise emission, shock waves, and micro jets [17]. Thus, when the leakage flow exits the blade tip gap, a flow jet can be produced [18]. Additionally, when the flow jet departs from the suction side and interacts with the freestream flow at a separation line, a tip vortex is produced [19]. As a result, the tip leakage vortex is made, which affects cavitation, flow instabilities, erosion, and noise [20].

Hydroelectric power plants have been investigated due to their capacity to adapt to changes in the use of electrical power networks by Alligne’ et al. [21]. Hydraulic machinery was subjected to off-design operation to keep up with this shifting demand. Hence, under specific circumstances, the swirling flow leaving the runner of a Francis turbine may operate as an excitation source for the entire hydraulic system. In fact, the main aim of this study was to determine how the location of the full load excitation source affected the stability of the system. Branko has carried out noise sampling, signal processing, and analysis as part of the vibro-acoustic diagnostics of turbine cavitation [22]. These were looked at in a series of prototype and model tests at several weak points in the practice. After that, new methods and advancements were made. These methods allowed for the early detection of negative impacts on turbine operation and the extraction of data on cavitation specifics. Nicolet et al. found that when a hydro turbine had to be turned off quickly, it put too much stress on the wicket gates and caused the head, discharge, speed, and torque to swing wildly, which led to breaking the safety pin [23]. Based on these findings, if the turbine shuts down for no reason, it could hurt the moving parts and the operating instruments that are connected to them. Two hydropower facilities’ runner blade deformation was examined by Gagnon and Leonard [24]. They reported that load rejection causes the most fatigue damage in both situations. Hydro turbine erosion caused by solid particles is also called sand erosion, silt erosion, or hydro-abrasive wear [25,26,27]. Moreover, mechanical wear caused by water sediment is a major global HPP operation and maintenance issue. Hydraulic turbine components exposed to sediment-filled water can wear out and cause sudden power plant shutdowns. In other words, the dynamic action of silt and water flow impacting on the solid surfaces of hydraulic components leads to this damage [28]. Runner blades, nozzles, guide vanes, ring liners, and inlet valve seals, etc., are those parts of the hydro turbine that are unfavorably influenced by residue [29]. Deterioration in the operation and functioning of HPPs because of the sediment effect causes catastrophic problems not only for turbine performance, but may also lead to complete collapse of the power plant. For example, vibration associated with mechanical wear might cause cracking and collapse in these power plants’ foundations and infrastructure. Additionally, as a result, the plant’s output efficiency gradually decreases [30]. In general, various factors influence the growth of hydraulic machinery’s sediment wear process, including particle mean velocity, mass, concentration of abrasive particles in a liquid flow, grain size, and particle collision angle on the surface of the turbine blades [31]. The documents available in the factories indicate that most of the hydraulic turbine parts are made of stainless steel with high hardness. Additionally, most types of hydro turbines include low-carbon super martensitic stainless steel 13Cr-4Ni-(Mo) rotor blades and guide vanes [32,33], but this does not protect the main parts from water sediments, particularly the runner blades and guide vanes. In this regard, three ways have traditionally been considered to overcome water sediment damage, including the design improvement of hydraulic components, development of a new wear-resistant alloy, and component coating [1]. A coating can be defined as a thin film formed or deposited on the surface of a component made of another material that is similar to the mechanical properties of the matrix and has good corrosion and wear resistance [34,35]. Surface coating technology is an effective way to enhance the silt wear resistance properties of different components of hydro turbines by modifying their surfaces. In this regard, hard metallics, carbides, borides, oxides, carbonitrides, metal-ceramics (cermet), silicide, and non-metallic materials have all been employed to resist wear damage in various coating systems. Such coatings can be made by reinforcing ready-to-use coating materials or by fabricating in situ coatings (by mixing multiple powder precursors in the desired ratio) with the help of a suitable heat source [1]. Under heavy silt conditions, the most preferable characteristics of a protective coating are cavitation, abrasion, erosion, and corrosion resistance; strong bonding to the substrate; vibration damping; and easy applicability at the site.

Despite conducting extensive research in the recognition and development of contemporary techniques to reduce the effects of mechanical wear in the damaged components of turbines, the current research describes some methods, allowing the deployment of coatings with nanochemical composition and extraordinary properties to improve functional performance. Nanostructured coatings have recently attracted attention due to the possibility of synthesizing materials with unique qualities such as high hardness, toughness, and wear resistance, which make them technologically attractive for a variety of industrial applications. This paper discusses the various types of wear to which mechanical equipment is exposed, as well as the technological methods to prevent it, especially the coating techniques with nanomaterial.

## 2. Structure of the Kaplan Hydraulic Turbine

A hydro turbine is a rotating machine that converts the energy of flowing water into rotating shaft energy. In other words, it is a wheel-like structure attached to the shaft, and the wheel begins to rotate when it is impacted by water flow [36]. Based on the fundamental method of utilizing water energy, turbines for small-scale hydropower projects are classified as either impulse turbines or reaction turbines [37]. Reaction turbines (e.g., Kaplan, Francis, and Archimedes Screw) are typically utilized in non-mountainous areas with relatively low heads and large flow rates. On the other hand, impulse turbines are designed for medium-to-high heads and low flow rate applications (e.g., Pelton, Turgo, and Cross-flow) [38].

Kaplan turbines are generally utilized across the world. In fact, the main idea of manufacturing the cost-effective and small Kaplan turbines was for individual use. Additionally, due to the large number of this type of turbine in industries, it is the main focus of the present research.

### Kaplan Hydraulic Turbine

**Figure 1:**
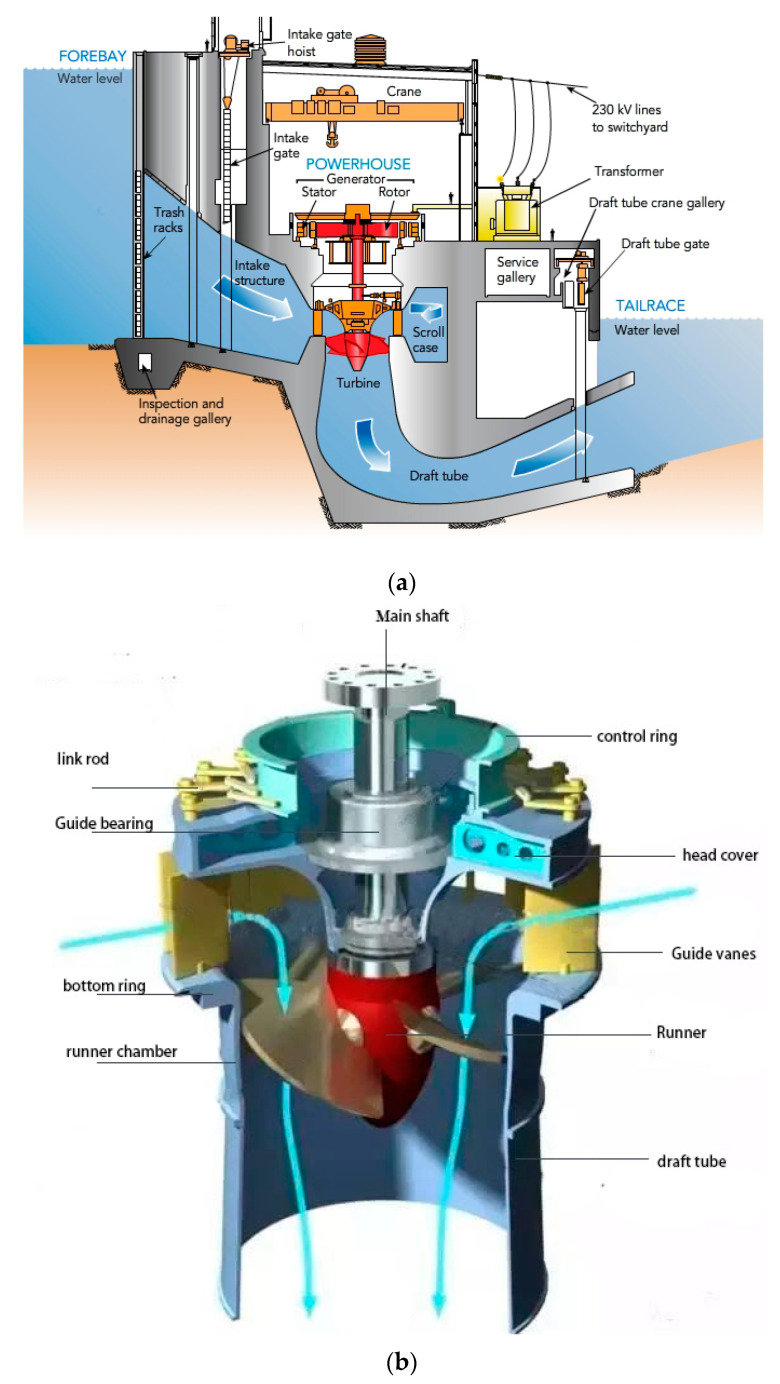
(**a**) Side view of the hydropower plant and (**b**) the main parts of Kaplan turbine [42].

The Kaplan turbine is classified as a reaction turbine, and it is a propeller-type water turbine that has movable edges; Viktor Kaplan, an Austrian teacher, invented it in 1913. The operational head of water ranges from 10 to 70 m and the power generation from 5 to 200 MW [39]. From its apparent head of 34.65 m, the Kaplan turbine foundation is said to provide the highest power, and each of the ten 4.8 m wide sprinters at the Tacoma Power Plant (Venezuela) will produce 235 MW. Kaplan turbines are currently more popular due to their dual regulating function, which allows them to operate under a variety of hydraulic circumstances. Kaplan turbines can save more time and resources in the construction of power plants than higher-head turbines since they have a smaller excavation depth and require less earth excavation [40,41]. Figure 1 depicts a side view of the hydropower plant and the main parts of the Kaplan turbine [42]. The main parts of the Kaplan turbine are: spiral casings; stay ring and stay vanes; guide vanes; runner blades; and draft tube.

All the mentioned components of a hydraulic turbine suffer from different variations in operational regimes, which lead to the turbine’s failure and even to a catastrophic consequence. Before starting to analyze different damages in hydraulic turbine, we have provided some different types of historical failures and their possible sources of origin or reasons of failure in Table 1. As shown in this table, the main reason why turbines fail, especially the moving parts, is due to how the turbine is being used. Most of the time, the main parts of turbines are made to be very resistant to wear, but that does not mean they cannot get broken. Additionally, cavitation is one of the main causes of turbine damage, especially in reaction type turbines, and usually causes the turbine to vibrate, which leads to fatigue cracking and runner blade damage. However, there are a lot of different cases that result in the failure of hydraulic turbines. These cases are also reviewed and discussed in Section 3 in the appropriate subsection according to the reason for the failure.

## 3. Various Damages in Hydraulic Turbines

In its simplest form, failure can be defined as any change in a machinery part or component that results in its inability to execute its intended function satisfactorily [58]. Machines are the core of every production line. Failure of the equipment carries a huge cost, which is not limited to wasting money and time on directly repairing equipment, but also includes the cost of unused equipment and the cost of lost unemployment benefits. To avoid excessive losses and limit business disruptions due to damaged machinery and equipment, it is necessary to investigate the causes of equipment failure. Machines fail for a variety of reasons. Some of these causes are due to material and physical defects, assembly, and manufacturing flaws, while others are due to external and operational factors. In the following, a detailed description of them will be given.

### 3.1. Internal Object Damage (IOD)

These types of damages are internal in the sense that an external factor does not cause the damage. Furthermore, diagnosing this type of damage is very difficult and must be conducted using special equipment and high experience. For example, the cavity created under the surface of the part due to the wrong casting process cannot be seen and recognized by the eye, and this damage can significantly reduce the strength of the part. In the following is a complete description of this type of defect in the field of turbine blades.

#### 3.1.1. Material and Physical Defects

A hydro turbine is an important part of any power plant that works at its best by keeping its operating conditions stable. After a few years of operation, hydro turbine performance and efficiency may decline for several reasons, one of which is material and physical defects. Material and physical defects are flaws in the raw materials that make up a product, such as problems with the supplier, problems at the time of delivery, problems caused by improper storage, and so on [59]. In addition, the handling and production of materials can lead to material flaws. Many deviations in physical characteristics are brought on by the presence of material flaws and impurities. Moreover, material flaws and impurities change the hardness and other physical properties in certain places. In this way, one of the most common worries is damage near the spot where the welds are being performed. Additionally, the welding process parameters will change the physical properties of the material in the welding area. Stainless steel is used in many parts of hydroelectric plants, especially turbines that must work in environments that are both corrosive and erosive. In this regard, the martensitic series are used to make parts with high mechanical strength and moderate wear and corrosion resistance. Additionally, the austenitic series are used when better corrosion resistance is needed, even though their mechanical strength is lower [60]. The stable chromium oxide barrier that forms on stainless steels makes them very resistant to wear and corrosion. For example, Francis and Kaplan turbines’ runners were made of martensitic (16% Cr and 5% Ni) steel, which contains a tiny amount of austenitic iron, up to 1972. Even though the wires were made of the same materials as the runners, some issues still appeared. The issue was that after normal cooling to 50 °C, where the transition from austenitic to martensitic should have taken place for the given composition of Cr/Ni steel, the weld compound was completely austenitic. The cause of this deviation was discovered because nitrogen (Ni) was added to the electrodes’ mantel to create a more flexible austenite weld deposit and reduce the risk of weld cracking during cooling after stress relieving up to 580 °C and then cooling to a martensitic [61,62,63]. Among all stainless steels, since 304 and 316 stainless steels have a lot of chromium and nickel, they are very resistant to destructive phenomena, such as erosion, wear, and corrosion. This is why they are widely used to manufacture blades for hydro turbines. In fact, 304 and 316 stainless steels are in the austenitic grade class. Austenitic grade stainless steels have a structure that makes them non-magnetic and prevents heat treatment from making them harder. Table 2 and Table 3 show, respectively, the chemical composition and mechanical properties of these stainless steels [64].

As an important part of a hydroelectric generating set, the turbine runner is not only the core of energy conversion, but also affects the hydraulic performance and reliability of the whole set. Large turbine runners are hard to move around because of traffic, so most of them are put together and welded at the unit’s installation site after each blade is made at the factory. High temperatures in areas near the welding zone can cause flaws in the metal used to manufacture the turbine’s runner blades because it is hard to perform heat treatment in the field [65]. Once the problems are bad enough, the turbine will start to shake more, and cavitation at the water outlet on the back of the blade will make the working face wear out faster. This significantly reduces the turbine’s power output efficiency. Additionally, the operation of the turbine unit poses significant potential risks to safety. Because hydraulic turbines are so big and it is hard to obtain data on faults, not many scholars have investigated how to find surface defects on turbine runner blades and other related topics.

A penstock rupture at a small hydropower facility in Poland in December 1997 is an example of this type of accident. The investigations on the material tests of the ruptured penstock shell revealed that the main structure of the material was pearlitic/ferritic with non-metallic inclusions. In addition, due to the decreased tensile strength (40–50%) of the welded seams in the penstock shell compared with the raw material of the penstock, as well as the tool delivering little heat on the material in the weld joints, it led to microcracks, gas bubbles, oxides, and cold droplets in the first fusion region of the raw material [66]. Moreover, it is claimed that if the material defects happen in the zone where the runner frequency coincides with the dynamic pressure, excitation frequencies may accelerate to the fatigue process, leading to blade vibration and damaging the blade [67]. Arrington has written about the crack in the steel penstock at the Oneida station hydroelectric plant and the break in the lower needle valve body at the Bartlett dam in the United States [68]. If the inspection is carried out at an early stage, the use of materials with early defects that lead to lower quality will be reduced. Failures of any part of turbo equipment usually start in a critical zone with a high concentration of stress, such as a metallurgical discontinuity or an area with a lot of wear. In any case, material defects in turbine parts will speed up the process of failure [8,69]. The auxiliary shaft of the 105 MW Kaplan turbine failed. This failed shaft was positioned inside the turbine runner and was connected to the turbine blades. Its primary duty was to turn the Kaplan blades in the flow direction to obtain maximum turbine efficiency. The hydraulic turbine had been operational for approximately 12 years [70]. The failure study revealed that the main reasons for the crack initiation and subsequent fracture propagation were a stress concentration placed near the failed shaft and frequent load changes. They proposed non-destructive inspection of the auxiliary shaft materials, considering variable material compositions, to detect cracks and material damage [70]. Material and physical defects are essentially controlled during the manufacturing stages of the turbine and its components, so that the manufactured turbine parts meet the standards required by the hydropower plant. However, it is necessary to preserve the properties of intact turbine components during the assembly and installation phases [71,72]. A key step in preventing damage during operation is choosing the right material for the turbine and the best way to make it. Typically, improper coatings and welding joints change the metal’s characteristics, which can cause material defects. After many reviews, it can be concluded that a big problem that can become a cause of turbine failure is the hydro turbine’s assembly, which is connected to several components and welding joints [73].

#### 3.1.2. Deficiencies in Design

Design flaws are undesirable characteristics of a product or system that arise as a result of the design process. This procedure includes the creation of the initial concept, the definition of the general configuration, and details in design (i.e., selection and specification of materials and manufacturing processes) [74,75]. Inadequate dimensions, such as thickness and radius, functional limitations, and a failure to anticipate service conditions, etc., are all examples of design flaws. Because these types of design flaws are frequently red herrings, the previous explanation on material faults also applies to them [76]. A part is not always imperfect just because it was designed and made with less-than-ideal features. Hydroelectric turbines are designed to transform the energy associated with moving water into mechanical energy. This is accomplished using a series of metal blades that are connected to a central shaft. As experience has taught us, we must teach the new hydro generation that many power plants have had problems and accidents, some of which have resulted in death and significant financial costs. A lack of effective design and review procedures, or in the case of small plants, lack of proper design, is one of the reasons for these incidents [77]. One of the accidents at a hydropower plant because of design flaws was at the Stugun hydropower plant, located in the north of Sweden [46]. Various tests were conducted due to the renovation of the generator and the turbine. Preliminary observations indicated that a reverse water hammer had broken the runner blade and head cover. To perform a comprehensive analysis, a reconstruction, redesign, adjustment, or enlargement design should be reviewed. An incident occurred during commissioning testing at the Akkats rebuild plant in Sweden in 2002 [46,78]. Although extensive repairs were made, it was only able to regain 80% of its generation capacity. During a load rejection, the guide vanes rapidly close. Therefore, water column separation collapsed and caused the lifting of the runner with 700 tons of shaft and generator rotor. In this case, all steps of design had to be conducted carefully again.

Tata power company’s Bhira installation was India’s first pump storage facility, opened in 2002 [46]. Because of its unstable properties, the pump turbine generated penstock resonance during trial operation, resulting in penstock rupture and the deaths of four individuals. Unexpected challenges may have been avoided if the above-mentioned design and review procedures had been followed [46]. A dangerous runner lifting occurred in 2003 at Tianhuangping pumped storage power station, Zhejiang Province, China, the first such reported incident during load acceptance [79]. At this time, unit No. 2 was synchronized and operated in a turbine mode for trial operation. In this plant, water was conveyed to six 300 MW reversible reaction turbines through two headrace tunnels of 7 m in diameter, which branched in front of the powerhouse into three penstocks with a diameter of 3.2 m. Each of the six tailrace tunnels had a diameter of 4.4 m. Connecting three 7 m diameter penstocks to one makes the hydraulic properties more unstable. Simultaneous load rejection is particularly hazardous; three units passing through an unstable zone at the same time can cause unanticipated surging [46]. It was not addressed in the feasibility study or general design; it should have been addressed in the detailed design (following bidding) and verified during commissioning and operation.

**Figure 2:**
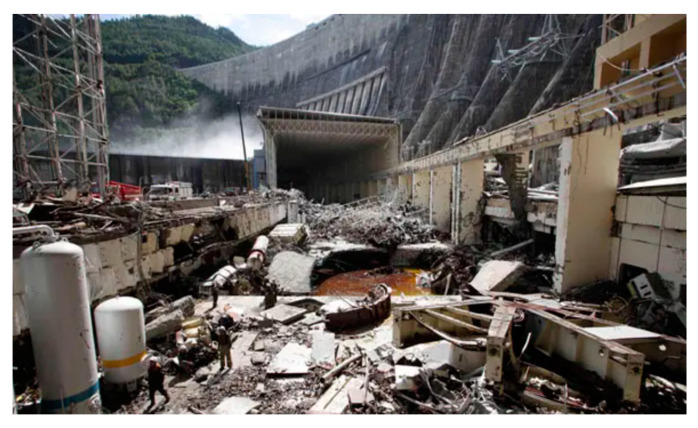
Sayano-Shushenskaya hydropower plant after accident [52].

Another major problem was Serbia’s Bajina Basta pump-turbine system. To achieve economic, social, technical, and environmental success, the design, building, and operation of any hydroelectric plant require numerous details to be effectively conceived, accurately executed, and carefully coordinated. After bidding, the highest head pumped storage station at the time was meticulously reviewed by the design team, and they also studied manufacturers’ data; eventually, all design stages were approved [51]. The Sayano-Shushenskaya power station catastrophe is one of the most serious hydropower plant disasters. The Sayano-Shushenskaya dam in Russia, near Sayanogorsk, collapsed catastrophically, as shown in Figure 2, flooding the turbine hall and killing 75 people [52]. Accordingly, turbine No. 2 was repaired from January to March 2009, and a new automatic control system was installed to slow or speed up the turbine to adjust output to changes in power demand [53]. It seems that this adjustment, modification in project design, and parameters were not applied in commissioning and trial operations [46,51,54]. Despite careful design procedures, the dangerous water hammer was not detected during the design stages; fortunately, preventing the machine from running into the unstable zone problem was achieved, but the system remains on the verge of disaster if the control system fails. The water hammer was the source of hydraulic force, which had ruined the turbines and generators as in an explosion. From a fluid mechanics and hydraulics perspective, the authors believe that reverse water hammer is a much more likely source of such a force.

#### 3.1.3. Deficits in Manufacturing and Assembly Processes

Kaplan turbines are commonly utilized in low-water head and large-capacity hydropower projects [71]. Various static and dynamic pressure loads are applied to Kaplan turbines. The net head and flow rate passing through the runner determine the static pressure load. Moreover, dynamic pressure load is created by the rotor–stator interaction or other less common dynamic phenomena such as vortex rope, tip vortex, and Von Karman vortices [70].

Manufacturing is a series of processes to convert raw materials into valuable products in the market. Manufacturing processes can be divided into two basic types: processing operations and assembly operations. There is a strong correlation between material selection and the manufacturing process. They could be said to be elements of the universal set called the Design Process. Casting, CNC machining, and forging are the most common fabrication techniques for hydro turbines. CNC machining technology is currently used by most major turbine manufacturers around the world. CNC milling, grinding, polishing, and static and dynamic balancing of the turbines are all part of the process. CNC machining is both performed on the body of the runner at once and on the blade/buck of the runner separately and then welded together with the other runner components [71,73,80,81,82]. Other manufacturing methods, including turning, forging, rolling, bending, and welding, are used to make the remaining components, e.g., spiral casing, head cover, guide vanes, and draft tube.

Manufacturing deficiencies are one of the most common causes of equipment failures that operate under normal operating conditions. They can occur when a product’s manufacturer utilizes the wrong material or when proper quality controls are not implemented at the manufacturing facility. A significant volume of blades, which are basically just cast, is causing a major manufacturing challenge. The stage of initial crystallization of the liquid metal, which causes internal tensions and alloying element segregation, is critical. The fatigue properties of stainless cast steel are substantially affected by casting defects, resulting in damage to the hydraulic turbine’s main components. For example, turbine runner fatigue properties have received a lot of attention in recent years. However, this renewed interest is due to a combination of factors. One of them is that power plant owners want turbines with a longer lifespan. Another reason is that continuous demand to increase turbine performance and the development of numerical methods tends to push new runner designs closer to the cutting edge of material limits, and this, in turn, demands a better understanding of materials’ behavior. Finally, the importance of cracking events is exacerbated by the pressure put on the plant operator to increase the availability of turbine-generator units. In the past, repairs could be conducted on a regular basis during scheduled downtime, but as this downtime becomes increasingly limited, there is less opportunity to repair cracks without production loss [83,84]. Due to the presence of welding discontinuity and high thermal stress, welded joints of turbine runners are one of the most critical sections of hydro turbines. In fact, temperature cycles, solidification, cooling distortion, and residual stresses can cause discontinuities of different types and sizes in welded joints. Mechanical assembly is another method that can be used to link two (or more) parts in a junction that can be disassembled easily. Traditional methods in this category include the use of threaded fasteners (i.e., screws, bolts, and nuts). The uncertainty of the mechanical assembly process usually brings great challenges to product quality control and assurance [85]. Some researchers believe that assembly process errors are a major source of defects and reduce product margins. In addition to the above, the most serious problem in hydropower facilities is equipment vibration, which is caused by mistakes in the mechanical assembly process. Failure of the equipment due to vibration causes shut down, and sometimes even a disaster in hydropower plants [86,87]. Additionally, one of the Kaplan turbine damages is rubbing. Due to the small tip, the blade tip may contact the stationary wall due to high radial forces, normally caused by unbalanced or fluid instabilities [88]. Unbalance can be of mechanical or hydraulic origin, and it could happen due to non-correct assembly of the rotating series, especially of the runner blades or the generator poles.

### 3.2. Foreign Object Damage (FOD)

Surface engineering is one of the most relevant current fields of research. The events that occur on the surface (i.e., wear, corrosion, cavitation, erosion, and stress concentration) create regions prone to crack nucleation, which under static or dynamic loading will eventually lead to component and structure failures [89,90,91]. FOD is the result of things being ingested into hydropower plants, and it is a great concern as it can lead to damage to the main elements of hydro turbines (where small debris and loose objects cause damage to manufactured equipment). It has been estimated that FOD costs the electrical power sector billions of dollars annually in damaged equipment and reduced efficiency of electrical power plants. In general, wear rate is determined by the geometry of the interacting surfaces, the type of interaction, material properties, load and surface pressure, ambient temperature, humidity, atmosphere, surface properties, and relative velocities between interacting surfaces [92].

#### 3.2.1. Corrosion Failures

Damage to hydropower plants is often the result of corrosion and mechanical wear. The loss of materials as a result of a chemical or electrochemical reaction with the environment is known as corrosion (materials suffer a loss of mass and strength when corrosion occurs). Corrosion has become a constantly evaluated cost that is assumed to occur, and it is always factored into production costs in many sectors [93].

**Figure 3:**
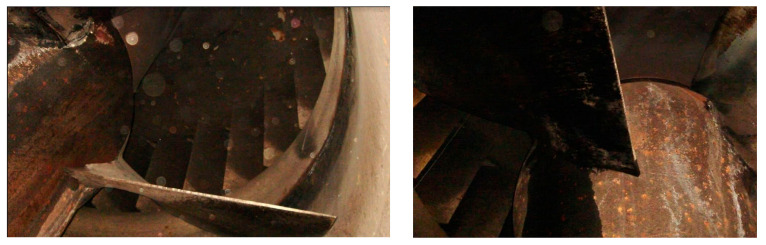
Failure due to the corrosion of Kaplan turbine.

The corrosion phenomenon is abundantly seen in industrial parts such as intakes, penstocks, isolation valves, scroll cases, wicket gates, turbine runners, draft tubes, spill ways, and radial gates. Figure 3 illustrates this destructive phenomenon in turbine blades that led to failure. Often, the first point of attack is at or near the Heat Affected Zone (HAZ) adjacent to the weld regions. The corrosion resistance of a material is perhaps the most critical aspect in determining whether it is suitable for a certain application or not. In practice, corrosion rate can be calculated using thickness data gathered at more than two separate times [94].

In the following, a detailed description of the most common types of corrosion, which is mainly related to hydropower plants, and especially the Kaplan turbine, is discussed.

##### Uniform Attack

**Figure 4:**
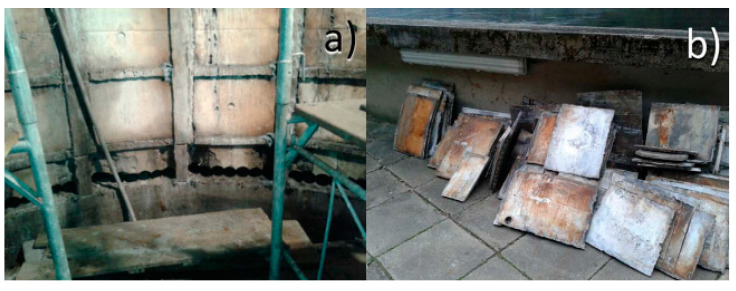
(**a**) Replacement of draft tube and (**b**) individual sheets after removal from the draft tube [98].

Uniform corrosion is a type of corrosive attack in which the damaged areas are evenly distributed across the attacked material. Because the assault happens throughout the entire exposed surface, uniform corrosion can quickly leave vast amounts of material unusable [95]. The corrosion of steel structures in freshwater flows is a significant concern in hydraulic engineering. The high level of dissolved oxygen in water, its hardness, and activity, which are governed by the hydrogen-ion concentration (PH), and the effect of the products of living fouling organisms’ physiological activity, determine the corrosion mechanism. The penstock is used to drain water from the source to the powerhouse hydro turbine. This is the most critical portion of micro hydro, because it transfers the water’s potential energy into kinetic energy [96]. Another essential part of the Kaplan turbine is the draft tube, which connects the water turbine exhaust to the tailrace. The water canal that transports the water out of the turbine is known as the tailrace. It transfers the kinetic energy of the water at the turbine’s outflow to static pressure and is normally found near the turbine outlet [97]. The most typical type of wear that attacks these sections of hydro turbines is uniform corrosion. Stainless steels, which are used to make hydropower plant components, are complex alloys that contain not only Cr and Ni as their main alloying elements, but also Mo, Mn, C, N, Ti, and other elements. These elements can precipitate in the form of secondary particles, such as carbides, nitrides, and sulfides, depending on their solubility. The existence of such secondary particles in the microstructure can have a significant impact on the final component’s mechanical characteristics and corrosion resistance [98,99,100,101]. An example of this is the damage to a 48 MW Kaplan draft tube at a hydropower plant in the Czech Republic. In this turbine, flow is entered through a spiral casing, passes through a runner, and finally leaves through the draft tube, which transforms dynamic pressure to static pressure [98]. The user inspects the draft tube, which is a steel weldment embedded in reinforced concrete, yearly. After a visual inspection indicated cracks, a non-destructive testing approach was used to validate their presence. During the repair process of the draft tube, every high-temperature activity (i.e., welding and grinding) produced fracture progression. Horynová et al. have performed a basic metallographic analysis, which revealed the presence of an intermediate phase that lowered the material’s ductility. Heat treatment has been used to eliminate this phase, but it is precipitated again during the welding process [98]. The failed parts of the draft tube were replaced as illustrated in Figure 4, and the failed material was sent to a laboratory for additional analysis to determine the cause of the failure and its mechanism.

After performing a comprehensive analysis of the chemical composition and studying the microstructure of the failed parts, they reported that the failure was due to intercrystallite corrosion resulting from unsuitable chemical composition and microstructure [98]. In this case, replacing the failed parts and using protective coatings is the most common solution for corrosion control. Isolating the reactive structural elements from environmental corrosives is the main function of the protective coatings, and these materials occupy a very small fraction of the total volume of a system.

##### Pitting Corrosion

**Figure 5:**
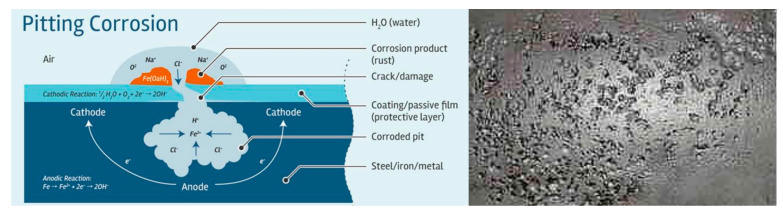
Details of the metal surface damaged due to pitting corrosion [103,104].

Pitting corrosion is a localized form of corrosion by which holes, or cavities, are produced on the metal surface as shown in Figure 5. Because it is more difficult to detect and anticipate, this type of corrosion is more harmful than uniform corrosion damage. Therefore, a small and narrow pit with minimal metal loss can lead to the complete destruction of an engineering system [102].

In general, when metal surfaces, such as high-strength stainless steel, are exposed to corrosive attack in severe environments, pitting corrosion can occur. This form of corrosion generally occurs when a small area is affected by the environment and becomes anodic. Meanwhile, a cathode is formed in another part of the metal. This causes a sort of galvanic corrosion that starts on the metal’s surface, but can progress downwards and finally lead to structural failure. This is especially true for dynamically loaded structures [105]. Intakes, penstocks, isolation valves, scroll cases, wicket gates, turbine runners, draft tubes, spillways, radial gates, and upriver dam nosing are all susceptible to this type of corrosion.

**Figure 6:**
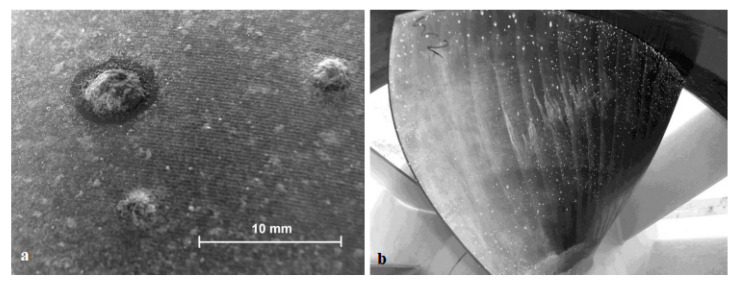
(**a**) The typical pattern of tubercles on a runner and (**b**) close-up view [106].

The hydroelectric power station Ashta-1 has 45 turbines and is located on the Drin River in Albania. Each turbine and its generator are housed in a module, with five modules forming a block with a shared water intake [106]. In this equipment, the Kaplan-type runner with a diameter of 1.3 m and the runner cap covering the shaft face are made of Nickel Aluminum Bronze (NAB) alloy, means CuAl10Fe5Ni5-C, according to EN 1982 [107]. Additionally, standard austenitic stainless steel 1.4301 is used for the runner ring (X5CrNi 18 10). Corrosion was discovered on most of the runners and runner caps only a few months after the turbines were installed. As shown in Figure 6a, tubercles of corrosion products up to 7 mm in diameter had formed, and there was some material loss beneath them, even though the material met the specification and followed the recommendation related to the ratio of aluminum and nickel content for optimum stability against selective corrosion. Figure 6a depicts the normal tubercle distribution on one of the runner blades, whereas Figure 6b depicts a close-up view. This is despite the fact that the runner rings were not damaged.

**Figure 7:**
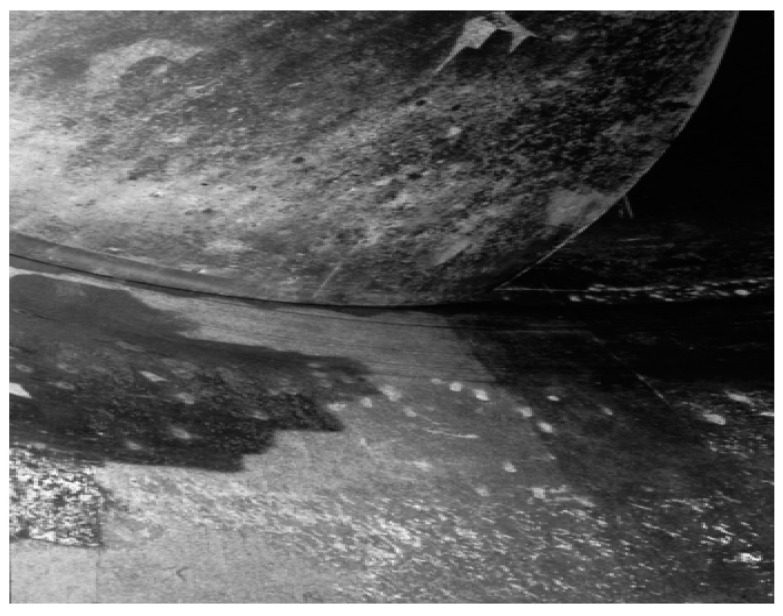
Deposits and pitting visible at a turbine blade and the runner ring in a hydroelectric power plant [108].

In another case, after only one and a half years of operation at a sound power plant on the Meuse near Roermond city, the Netherlands, pitting corrosion appeared on the four Kaplan turbine blade surfaces, made of austenitic stainless steels, as illustrated in Figure 7 [108,109]. The most common pitting corrosion causes are cracks in the surface layer, scratches and small chips, non-uniform stress, defective metal substrate, turbulent fluid flow, non-uniform protective coating, and chemical attack on the protective coating. In other words, these are the main causes of failure.

#### 3.2.2. Fatigue Failure

Fatigue is a failure mechanism that involves the cracking of materials and structural components due to cyclic loading (e.g., stress, strain, deformation, and thermal, etc.) [110]. According to Murakami and other scholars in the field of failure analysis of industrial components, fatigue is responsible for 80 to 90% of fractures [111,112,113,114,115,116,117,118,119], and fatigue cracks are most formed at geometrical discontinuities such as machining holes, notches, slots, gross-sectional transitions, and so on [120]. Fatigue cracks start at the location of the highest local stress/strain in the component and are almost always superficial before growing inside the component. In cases where there is a notch in the initial geometry of the part, this will usually be in a notch position. Moreover, the simultaneous effects of macro-notches and flaws (e.g., cracks) significantly affects the stress/strain concentration [121]. A crack’s “lifecycle”, which culminates in fatigue failure of a cyclically loaded component, is divided into five stages: crack initiation, microstructurally short crack propagation, mechanically/physically short crack propagation, protracted crack propagation, and final structure fracture [122]. Since most industrial parts are under the effect of dynamic and repeating loads, the occurrence of fatigue phenomenon in the industry is inevitable [123,124,125,126,127,128]. In this regard, large industries such as petrochemical and power plants are not excluded. Accordingly, material fatigue is a turbine failure mode. The turbine components that are subjected to alternating or cyclic stress below their yield strength fail progressively by cracking [82]. For this reason, the fatigue properties of turbine runners have received a lot of attention in recent years. Runner blades experience increasing dynamic strains due to the greater operating range of turbines due to changes in the use of electrical networks [129]. Since blade fatigue cracking is one of the predominant degradation mechanisms, designers need to ensure that the runner will endure its expected lifespan without cracking. On the other hand, as numerical tooling improves and the demand for improved turbine performance grows, new runner designs are being pushed closer to the materials’ limits. In addition to the above-mentioned points, the demand on the plant operator to maximize the availability of turbine-generator units exacerbates the importance of cracking incidents. Repairs could formerly be conducted on a regular basis during scheduled downtime, but as this downtime becomes increasingly limited, there is less opportunity to patch cracks without affecting production [81].

The most important factors contributing to the decrease in fatigue are likely to be constructive, technological, and operational. These factors are as follows [130]:

- Constructive factors—shape and dimensions of the part and assembling method.
- Technological factors—material and the surface quality.
- Operational factors—loading type, short-term overloads and underloads, jerks, load frequency, temperature, and chemical influence of the environment.

**Figure 8:**
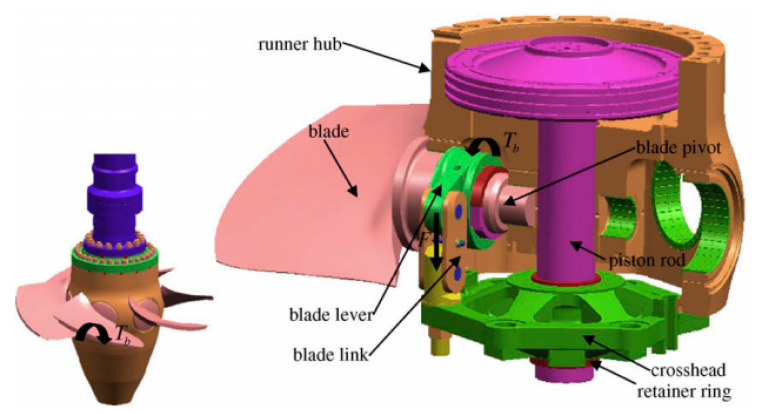
An overview of single blade control system [8].

Kaplan turbines have numerous blades in their runner that can adjust their incidence flow angle to provide good performance at all operating positions. To reach this goal, a complicated control mechanism placed inside the hub and the shaft changes the angle, as shown in Figure 8.

**Figure 9:**
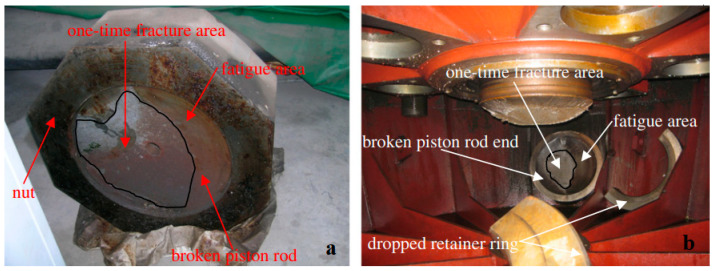
Fractured piston rods: (**a**) nut structure and (**b**) retainer ring structure [8].

In November 1995, production of a special turbine started: a 204 MW rated output Kaplan turbine ZZA315-LJ-800 moveable propeller with a specific speed of 22.6, an 8 m runner diameter, a 47 m rated head, and a rated speed of 107 rpm at Shuikou hydropower plant (Minjiang river in Fujian province, China). After starting up, the turbine was observed to be badly vibrating on 10 February 2000 [131]. Additionally, the oil level in the oil collection groove decreased greatly. Upon opening the housing, the central axis of the piston rod was found to have broken at the joint of the M540 nut with the crosshead falling into the cone, as shown in Figure 9a.

The nut structure was replaced with a retainer ring structure after the accident, but five years later, on 16 March 2006, the retainer ring structure similarly failed at the same position as that demonstrated in Figure 9b. A comprehensive investigation was performed, including dynamic analysis of position rods to identify the design problem [131]. A detailed analysis of the two cases revealed that the fractured areas exhibited fatigue failure due to cyclic loads. The most probable reason was the blades’ unsteady pressure loads. As they convert fluid energy to mechanical energy, the blades are subjected to tremendous hydraulic pressures. The hydraulic pressure on the blades causes torque on the blade pivot, which is transferred to the other parts of the system in the blade-control system. Furthermore, the blade torques are uneven and unsynchronized across the board [8,131].

**Figure 10:**
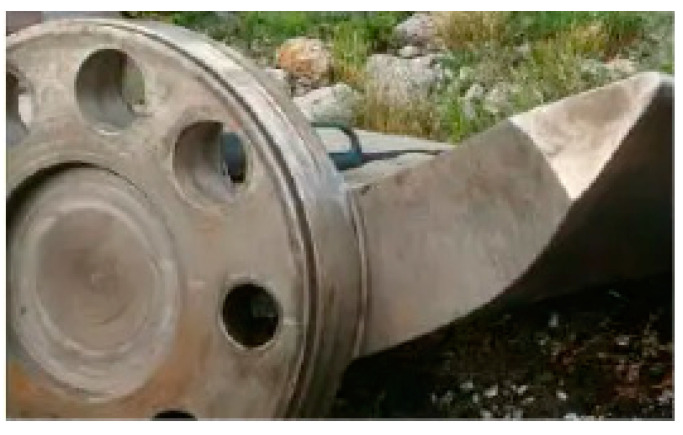
The cracked runner blade of a 35.5 MW Kaplan turbine in Romania [132].

Another case of fatigue failure involved a 35.5 MW Kaplan turbine runner blade in Romania. The damage occurred, as shown in Figure 10, in one of the turbines from the hydropower plant’s cascade, which worked at a higher head than the others, according to the investigation of the operating conditions [132]. The metallographic observations and calculations conducted led to the conclusion that the cracking of the blade began and developed from the stress concentration located between the blade and the blade flange in the leading-edge direction.

In summary, material, structure, loading, and operating circumstances are the four categories of parameters that determine the fatigue life of hydro turbines. However, other forms of failure modes, such as cavitation, erosion, and swallowed bodies, are briefly discussed to expose the comprehensive failure mechanisms in hydro turbines.

#### 3.2.3. Cavitation Wear

**Figure 11:**
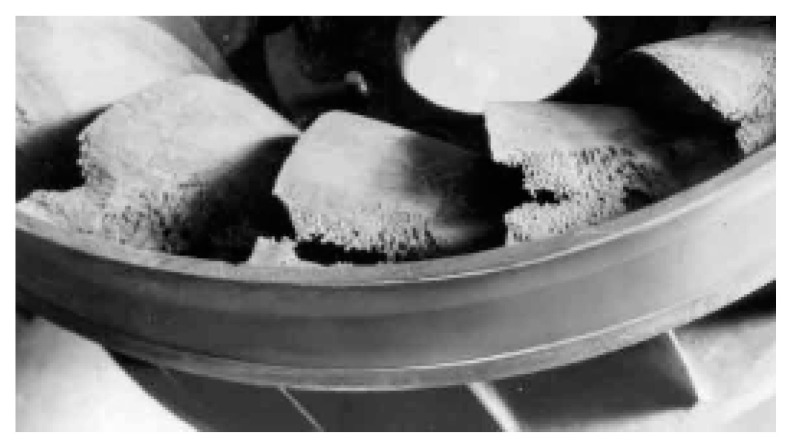
Damage in turbine part due to cavitation phenomenon [134].

Cavitation wear is the process of progressive degradation of a material due to the repeated nucleation, growth, and violent collapse of cavities in a liquid flowing near the material. Cavitation in hydraulic machinery has undesirable effects such as flow instabilities, excessive vibrations, machine performance loss, noise, material damage, and other problems [13,133]. Cavitation causes excessive pressure pulsations, damaging the runner and turbine channels. As a result, the water turbines’ total operating efficiency drops, and repair expenses rise. It is possible when vapor bubbles are formed in a liquid at a constant temperature. Bubbles grow if the pressure falls below the liquid’s saturated vapor pressure at the same time. The destructive phenomenon of cavitation has been an important issue in the reaction turbines for decades, which must be considered in the design stages (e.g., Figure 11 demonstrates the damage in turbine parts due to the cavitation phenomenon [134]). The runner blade design influences cavitation inception and development, as well as the operating conditions such as the machine setting level [135]. Accordingly, the most common types of cavitation in a Kaplan turbine are as follows.

##### Leading Edge Cavitation

**Figure 12:**
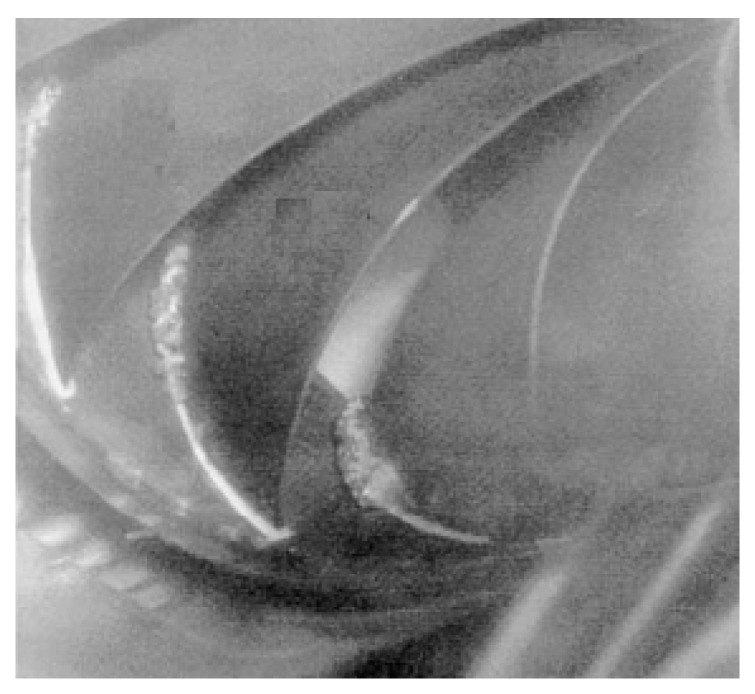
Leading-edge cavitation in the Francis turbine [137].

Among the cavitation types that may develop in a flow around a lifting body, so-called attached cavitation or leading-edge cavitation is known to be responsible for severe erosion [136]. It is a very common and complicated type of cavitation that can present different regimes depending on the hydrodynamic conditions. This type of cavitation is usually seen attached to the runner blades’ suction side, as shown in Figure 12. They are formed under operating conditions of high head and inflow incidence angles that are significantly larger than the design values. Due to the high-pressure difference between the pressure side and suction side of the runner blade, a leakage flow departs from the high-pressure region to the low-pressure region. The velocity of the leakage flow is particularly high and results in a decrease in pressure at the tip clearance. When the pressure drops below the saturation pressure, tip clearance cavitation generally occurs [18].

##### Tip Vortex Cavity Phenomenon

**Figure 13:**
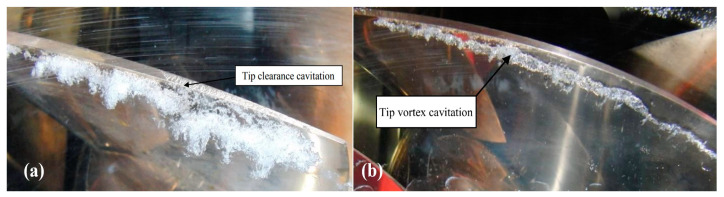
Different types of tip-related cavitation, including (**a**) tip clearance cavitation and (**b**) tip vortex cavitation [138].

This type of cavitation takes place at the tip of the blade, as shown in Figure 13a. The pressure difference between the intake and suction side of the blade causes flow through the tip clearance. The velocity of the water in the gap could be very high, which is linked to the decrease in pressure. Based on the technical reports of experts in this field and scientific achievements, if the pressure drops below the saturated vapor pressure, a tip clearance cavitation occurs [138]. Another type of cavitation related to tip clearance is called tip vortex cavitation. When the tip clearance flow leaves the gap, a jet is created. The jet leaves the suction side of the runner blade and creates the tip vortex. The tip vortex begins near the blade’s leading edge, detaches the suction side surface of the blade, and continues downstream along the runner blade as shown in Figure 13b.

**Figure 14:**
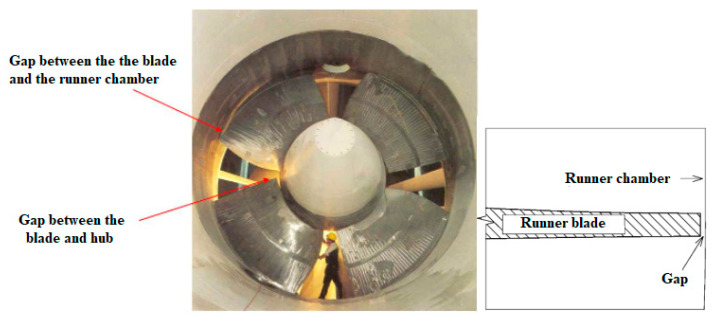
Gap between the runner blade and the runner chamber [138].

The Kaplan turbine’s optimal operation is ensured by a twin regulation mechanism. On-cam operation is possible because of the adjustable distributor and runner blade openings. The runner is not shrouded since the runner blades are changeable. This indicates that there is a space between the revolving hub with the blades and the stationary runner chamber [138]. Space is also known as a “tip clearance” (see Figure 14). In this regard, scientists have investigated the tip-leaking vortex that forms in the clearance between the rotor and the stator of axial hydro turbines. However, many related phenomena remain unknown. For example, it is still unknown how the clearance size relates to the incidence of cavitation in the vortex, which can cause severe erosion and damage to turbine blade [139].

The wake of the distribution guide vanes provides a very non-uniform pressure field in the Kaplan turbine area, resulting in repetitive collapses and rebounds of the cavitating tip vortices. Cavitation erosion is obviously influenced by the vortex’s strength and core size, as well as its course and distance from solid barriers.

**Figure 15:**
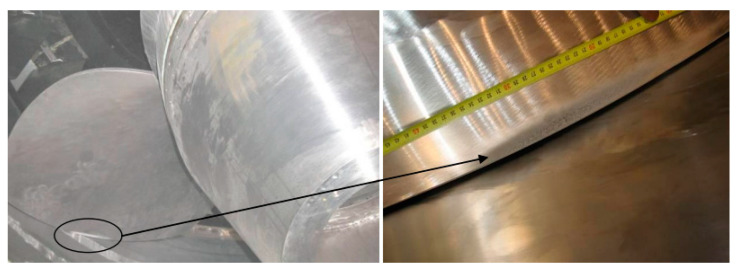
Cavitation pitting on the suction side of the blade near the runner chamber [138], the right image shows the part highlighted in the left one with higher magnification.

A cavitation was identified at a 10 MW rated power and 10 m water head Kaplan turbine. The dimensions of the cavitation were approximately 200 × 20 mm with a maximal depth of 3 mm located at the suction side of the blade near the runner chamber, as demonstrated in Figure 15. The CFD analysis revealed the presence of a tip vortex with a specific shape and intensity, which might generate cavitation pitting in the same location as the prototype.

##### Traveling Bubble Cavitation

**Figure 16:**
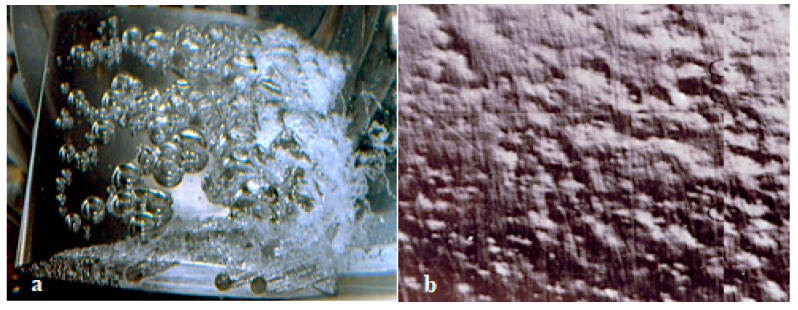
(**a**) Traveling bubble cavitation and (**b**) pit overlap and damage due to bubble cavitation [141].

This type of cavitation appears in the form of bubbles that move along the solid body. When the bubbles reach the vicinity of the low-pressure point, they become visible [140]. It takes the form of separated bubbles attached to the blade’s suction side near the mid-chord next to the trailing edge, as shown in Figure 16a. On the suction side of the blades, traveling bubble cavitation can be seen at the rated discharge. This is a severe and noisy type of cavitation that significantly reduces the machine’s efficiency and that can provoke erosion if the bubbles collapse on the blade [138,140]. Traveling bubble cavitation depends upon the water quality. As shown in Figure 16b, each cavitation bubble may leave a pit on the wall as it collapses.

Vibration is one of the most dangerous effects of cavitation. In addition, cavitation intensity is related to the amplitude of frequency peaks. Because the vibration frequency is inversely proportional to mass, the vibration amplitude decreases as the load increases. Additionally, when the turbine speed exceeds its design speed, the amplitude increases and the turbine performance decreases.

Due to the difficulty of repairing hydropower units, monitoring systems that detect cavitation during operation and aid in avoiding harmful circumstances appear to be the best option [138]. These cavity phenomena can be reproduced and visually observed during model testing in a suitable environmental laboratory. However, the conditions of apparition and the intensity of their unwanted consequences cannot yet be precisely scaled to the relevant prototype. As a result, unforeseen cavitation difficulties can occur during typical Kaplan turbine operation. Therefore, to detect and avoid them, appropriate detection techniques must be utilized, which may be simply and successfully implemented in real hydropower plants. The use of vibrations and pressures to detect cavitation seems to fulfill these requirements [142].

##### Hub Vortex Cavitation

**Figure 17:**
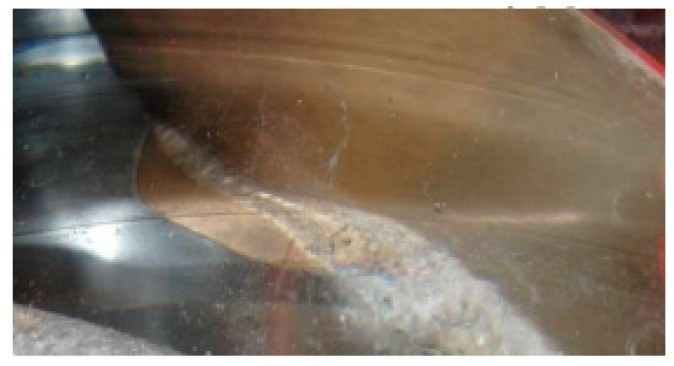
Extension of hub cavitation around the Kaplan turbine [144].

This is a sub-category of vortex cavitation [143]. It is observed in the central parts of vortices gyrating away from the liquid flow around the obstacle. The hub vortex cavitation is usually due to a high angle of incidence between the direction of water flow and the blade’s leading edge. It can result in the outer edge of the blade looking a bit moth-eaten. Because of the designed operating range, it usually appears at the runner’s hub, as seen in Figure 17.

As explained before, cavitation causes erosion, vibration, machine efficiency loss, and noise in hydraulic turbines, depending on the causes of occurrence, such as higher or lower head than the design step, partial load, or excessive load, and the velocity component of flow discharge. Even though cavitation erosion has received a lot of attention, accurately predicting the severity and rate of cavitation erosion in hydraulic turbines is difficult. In fact, cavitation phenomena are still a challenging process to understand because they involve hydrodynamic laws that govern the creation and collapse of vapor cavities, as well as the material’s response to repeated pressure pulses induced by these collapses [145].

In summary, the main objective of cavitation testing and inspection is to determine the risk level to overcome any efficiency changes and reduce erosion damage to the hydro turbines. In other words, each type of cavitation is evaluated in terms of its dependence on erosion risk [146].

#### 3.2.4. Hydro-Abrasive Problem

**Figure 18:**
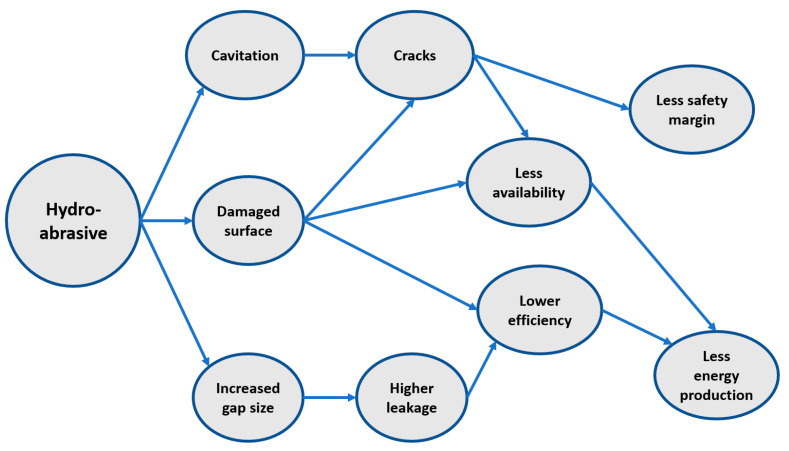
Hydro-abrasive effects on hydro turbines.

The flow of sediment particles in rivers is a major challenge for developing hydropower plants across sediment-laden rivers. Hard particles such as quartz and feldspar can be found in abundance in rivers around the world. The abrasive effects of these particles cause damage in hydro turbines, particularly in high- and medium-head hydroelectric power plants. Due to maintenance expenses and output losses, this has become a severe economic issue [147]. Hydro-abrasiveness is due to suspended sediments in the water that pass through the turbine, and depending on the impact conditions, the particles, which are harder than the surface material of the turbine parts, damage the surface. The surface material is worn away, resulting in geometric changes and efficiency loss, cavitation and mechanical problems, higher maintenance costs, less availability, and loss in energy production. Figure 18 depicts the above-mentioned cases. Abrasion in hydro turbine components is complicated, and it is governed by several factors: (1) eroding particle properties such as concentration, size, shape, hardness, mineral constituent proportion, and toughness, all of which have a direct impact on turbine material; (2) turbine base material; and (3) operating conditions such as velocity and impingement angle [4,5].

**Figure 19:**
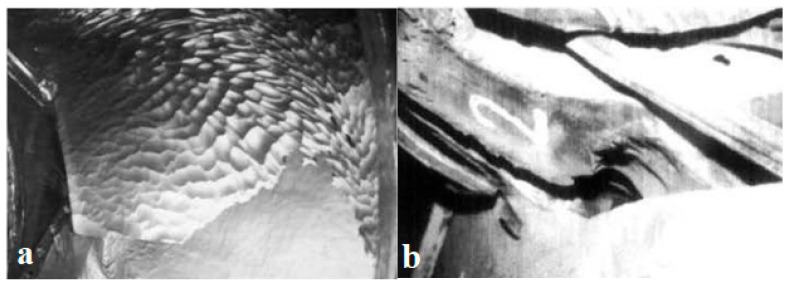
Extensive abrasion on Kaplan turbine components, including (**a**) runner blade and (**b**) guide vanes [26].

The Chilla hydroelectric project (India), located upstream of Haridwar in the Himalayan foothills, is a run-of-river scheme on the Ganga (this project was completed in 1980–1981). In this facility, there are four Kaplan vertical shaft turbines, each with a capacity of 36 MW and a design head of 32.5 m at the speed of 187.5 rpm [26]. Water is diverted to a 14.3 km long, 565 m^3^/s capacity lined power channel via a diversion barrage with a head regulator located 5 km downstream of Rishikesh town. Turbine components have been exposed to severe abrasion and erosion since the power plant was opened [148]. The high concentration and large size of silt particles from the Ganga cause extensive damage to the underwater parts of hydro turbine units, cooler tubes, drainage pump impellers, and valve seats. In the early years of operation, the blades of the Kaplan turbine were found to be extremely eroded and cracked on the trailing side after a few monsoon seasons [148]. Figure 19 shows extensive abrasion on the runner blades and guide vanes, which require heavy maintenance and major repairs.

#### 3.2.5. Hydro-Erosion Problem

**Figure 20:**
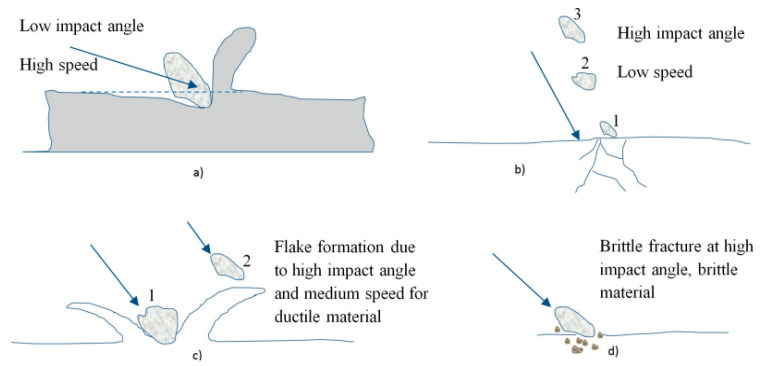
Various forms of erosive wear mechanisms, including (**a**) abrasive/cutting erosion, (**b**) fatigue erosion, (**c**) plastic deformation, and (**d**) brittle fracture [154].

Erosion can be classified under one of several types of wear caused by the impacts of solid and liquid particles on a solid surface. In fact, the flow contains particles that have enough kinetic energy to corrode the metallic surface. The mechanism of erosive wear is quite similar to that of abrasive wear, but in the case of abrasive wear, the eroding agent is much larger in size and the contact angle is smaller [28]. Surface roughening is the initial stage of erosion followed by pit formation. This leads to a significant reduction in the weight of the material [17]. In addition, the surface damaged by erosion makes the condition favorable for cavitation, which means a coalesced effect [15]. Weight loss in turbine components due to erosion depends on the load of particles entering the power plant. Two distinct erosion mechanisms arise from the impingement angle of a particle on the hydraulic surface. Impact damage occurs when a particle approaches the normal surface, causing the surface to crack at first, then loosen with repeated impacts, and finally excavate, as the surface is already cracked and loosened by the particle and removed by another impacting particle. Additionally, similar to mechanical grinding, a particle approaching parallel to the surface would scratch and gouge the surface [149,150,151]. Erosion damage is commonly defined as the slow disappearance of material produced by repeated deformation and cutting activities [152]. When particles strike the surface at a small impingement angle, the material is removed by the cutting mechanism [153] as shown in Figure 20 [154]. Moreover, the abrasive grits roll and slide when they strike on the surface at a small impingement angle and cause erosion by abrasion or cutting mechanism [154,155,156].

**Figure 21:**
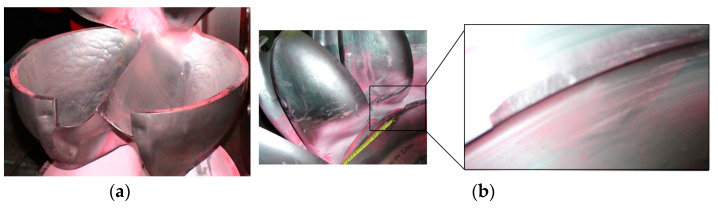
Sediment erosion in different parts of hydro turbines, including (**a**) surface erosion Pelton runner [158] and (**b**) damage to runner disk [159].

Various studies have been performed in each of the above mechanisms. Overall results show that the average velocity of particles, their mass, the concentration of abrasive particles in fluid, the size distribution of particles and their average grain size, the shape of the particles, the angle of impingement, the time interval of the attack, and the erosion resistance of structural material all influence the erosion damage process [157]. Silt erosion is designated as abrasive wear. This type of wear breaks down the oxide layer on the flow guiding surfaces and partly makes the surfaces uneven, which may also be the origin of cavitation erosion (see Figure 21).

**Figure 22:**
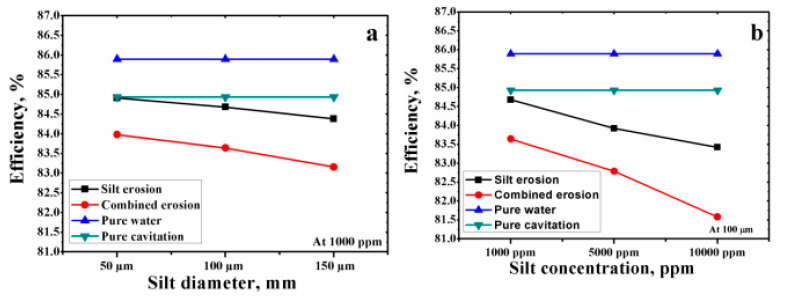
Efficiency trend of software modifications under pure water, cavitation erosion, silt erosion, and combined erosion at various silt parameters, including (**a**) silt size and (**b**) silt concentration [160].

Dinesh and Bhingole have calculated the efficiency of the Kaplan turbine considering different flow conditions such as particle size and silt concentration [160]. In this regard, overall efficiency of software modifications under pure water, cavitation erosion, silt erosion, and combined erosion at various silt parameters is demonstrated in Figure 22. In pure water flow, turbine efficiency is about 85.90% at full opening of the wicket gate. Moreover, in cavitation erosion operation conditions, the efficiency is reduced by 0.96% compared with that of pure water flow. Additionally, the efficiency of silt erosion operations decreases as the silt parameter increases. In this case, maximum efficiency drops of 2.47 pct were observed at a silt diameter of 100 μm and a silt concentration of 10,000 ppm. However, in combined erosion, the operation efficiency is drastically reduced compared with the other operation conditions. In combined flow with the same silt parameter, the maximum efficiency drop was 4.31%.

In addition, Weili et al. have applied numerical simulation to investigate the characteristics of cavitation of Kaplan turbines in pure water and solid–liquid two-phase flow [161]. They studied the effect of different concentrations and diameters on velocity, pressure, and particle concentrations’ distributive regularity on turbine blade surface. It was revealed that, by increasing the sediment concentration and sand diameter, the runner abrades more seriously, resulting in decreased turbine efficiency. The authors also concluded that the runner cavitation performance decreases with the increase in sand concentration and diameter of the particle. Consequently, this leads to a great decrease in turbine efficiency. Sangal et al. have analyzed the effect of different silt parameters on the Kaplan turbine’s efficiency [6]. They analyzed the regions with high velocity and pressure by applying CFD simulation. It was shown that silt erosion mainly affects the blade tips and region around the trailing edge. This is related to the additional hydraulic load and high velocity due to the circumferential speed and gap flow, respectively. The authors stated that silt erosion’s near to best efficiency point is the main reason for the maximum loss of Kaplan turbine. Moreover, it was confirmed by Thapa et al. [4], Man [5], and Mamata and Saini [157] that turbine blade erosion depends on different parameters such as velocity, operating conditions, impingement angle, surface hardness and morphology, substrates, elastic properties, chemistry, the shape and size of the particles, concentration, and hardness.

## 4. Practical Solutions

There are many practical solutions proposed by different researchers to prevent Kaplan turbine failure in terms of different types of failures. As it is related to the materials’ defects, soft martensitic stainless steels are commonly used for the manufacturing of hydraulic turbine parts, especially the runner blades [73]. The turbine blades are welded using 410 Ni Mo filler alloy [162]. Moreover, a post-weld heat treatment is required for this type of filler alloy to temper the brittle martensite formed in the welded zone and to reduce residual stresses induced by the welding process [163,164]. Stainless steel has become almost exclusively the best material for new projects and modifications of hydraulic turbine runners throughout the years. Because these materials offer a combination of exceptional weldability, high strength, and cavitation resistance, martensitic grades of stainless steel, including 13–16% Chrome and 3–5% Nickel, have been used to a great extent. Austenitic stainless steel has high cavitation resistance and weldability, but its strength is lower compared with that of martensitic stainless steel. Because of the higher chrome content, austenitic steels (18–20% chrome and 3–12% nickel) offer better corrosion properties than martensitic steels. Eventually, during the manufacturing and handling of any stainless steel, precautions should be taken to avoid contamination. To analyze failure and identify its main causes in industrial parts, it is necessary to research all the possible causes, and in some cases, in addition to using evidence, the answer can be obtained by rejecting the possible causes [11,165,166,167,168].

Additionally, good design of equipment should reflect the high performance and efficiency of the equipment under difficult operational conditions. In this regard, considering the past and present situations is very important for the future development of a structure [169]. For instance, in the case of Serbia’s Bajina Basta pump-turbine system, the design team discovered a dangerous instability by applying manufacturers’ data, and it was published for the first time [170]. In this regard, the control system has been modified to protect the system. The phenomenon was confirmed and published during the trial operation [171]. In addition to the design and development of low-head hydropower turbines, the researchers have also paid attention to economic considerations [172] and control and energy storage [173]. It is also reported that enlargement, redesign, adjustment, and modification in project design, as well as a special titled Hydraulic Transient Analyses (HTA), should be analyzed to avoid disaster [52]. All turbine parts must be designed and constructed to safely withstand maximum stresses during normal operation, runaway, short circuit conditions, out of phase synchronization, and brake application. In this issue, the maximum unit stresses of the rotating parts shall not exceed two-thirds of the material’s yield point [67]. In normal conditions, the safety factor based on the yield point shall not be less than three for other parts. Additionally, for over-load and short-circuit conditions, a safety factor of 1.5 on the yield point shall be permitted [174]. Reviewers who understand the complexity of the project have avoided disasters and problems, mitigated additional funding, deficiencies, and unforeseen casualties, and optimized the project design. It is also proposed that the design process should include an interphase between material selection and the manufacturing process for successful product design [80]. The complicated geometry of water turbine blades necessitates the use of specialized manufacturing techniques. Because of the curved three-dimensional surface geometry of large hydro turbine blades, deformation is one of the most common flaws during casting. To overcome this problem, inverse deformation is commonly used to achieve the desired shape for sand molds. On the other hand, it is difficult to estimate the amount of inverse deformation for different geometries, and in some cases, it is impossible for complex geometries [175]. Providing a safe, reliable, and healthy work environment is a requirement for organizations all over the world, and the best method to achieve this is to eliminate the dangers that are impossible in continuous process plants and manufacturing units. Quality assurance plays a key role during the deployment of any new project. Before being installed and physically accepted in the receiving department, all parts, equipment, controls, and other system components are evaluated based on the different engineering standards. In addition, before accepting an item from a vendor, material test results, certificates of compliance, and equipment test reports are all assessed and archived. Similarly, the systems are examined for potential flaws in accordance with engineering standards, internal instructions, and general regulations [176]. Regarding corrosion, some researchers have suggested manufacturing turbine blades from 13Cr4Ni stainless steel and then heat treating them at high temperatures by quenching and tempering [177]. In this study, the quenching temperature was about 1050–1080 °C. Moreover, the tempering temperature was in the range of 600–650 °C. Afterward, a regulated cooling process after tempering was carried out in the furnace to reduce the possibility of inner stresses and blade deformation. The results of material engineering research proved that this type of steel has a good corrosion resistance in moderately corrosive environments that are free of chlorides.

**Figure 23:**
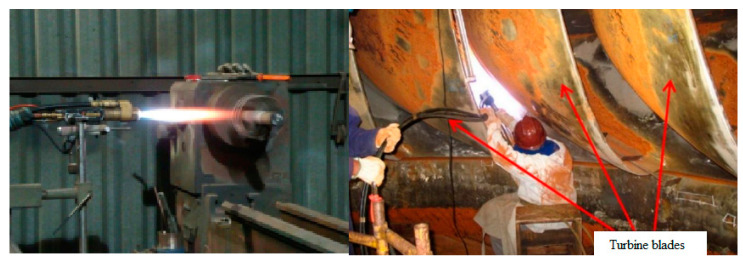
Alloy coatings deposited by the HVOF process [180].

A common technique for the repair of hydroelectric runners is metal replacement or coating, which does nothing to break the natural cycle of corrosion. Coating is a unique way to modify the surface properties of any substrate to suit an aggressive working environment for efficient work [178]. In other words, coating is a layer of material that forms naturally or is deposited synthetically on the surface of a component made of another material with the goal of improving mechanical properties and corrosion resistance [178]. To improve the corrosion resistance of component surfaces, different surface engineering techniques, i.e., thermal spraying, laser cladding, Physical Vapor Deposition (PVD), Chemical Vapor Deposition (CVD), hardening, and plasma nitriding, have been developed. Thermal spraying technologies such as detonation guns, plasma spraying, High Velocity Oxy-Fuel spraying, and cold spraying have already been used commercially on various equipment components [179]. Due to their combination of excellent toughness and high hardness, which are required for corrosion and mechanical wear resistance, the deposition of carbide coatings by the HVOF thermal spray process has been widely discussed in the literature and applied in the industry, as shown in Figure 23.

**Figure 24:**
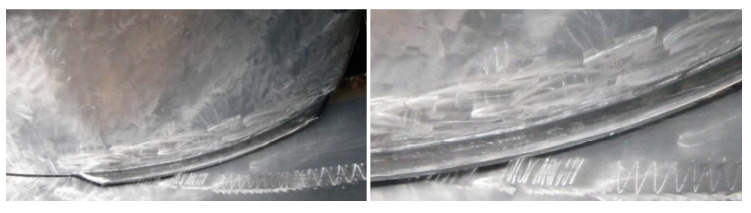
Kaplan runner equipped with anti-cavitation lips after 1500 operating hours [138].

Furthermore, WC–Co (powders with a bimodal size distribution of WC particles have been used to produce a coating by HVOF spraying) coatings have exceptional hardness and excellent toughness thanks to WC particles and Co binders [179,180,181,182,183]. WC–Co coatings, especially with nano-sized WC particles, have already been successfully utilized in wear-resistant equipment [184,185]. Recent studies have successfully demonstrated the simultaneous improvement of toughness and hardness of nanostructured WC–Co coatings. Nanotechnology helps in the production of various structural materials with various qualities, lighter weight, stronger composites, and self-cleaning surfaces. Therefore, instead of using conventional coating, nanopowder appears to give better results. New activities are being undertaken to investigate the performance of ceramic nanomaterials such as alumina (Al_2_O_3_), titania (TiO_2_), zirconia (ZrO_2_), and silicon oxide (SiO_2_) to improve the performance of steel against corrosion and mechanical wear. These materials are being used as reinforcements in other materials to prepare thermal spray composite coating compositions [186,187]. Furthermore, despite their excellent properties such as higher hardness, higher wear resistance, lower corrosion rate, and good wettability, thermal spray deposition of alumina and titania composites has received less attention [188]. Additionally, little literature is available showing the mechanical blending of nickel with alumina and/or titania nanostructured powders [187]. A coating must provide a continuous barrier to a substrate, and any flaw might become a focal point for substrate degradation and corrosion [189]. Nanoparticles have recently been used to improve the chemical, mechanical, material, and metallurgical properties of coatings. Nanocoatings are made up of nanoscale ingredients or layers that are less than 100 nanometers thick. Nanocoatings are efficiently utilized to reduce the influence of a corrosive environment due to their numerous characteristics, such as surface hardness, adhesive quality, long-term use, and high-temperature corrosion resistance, and to increase tribological properties, among others [190]. Moreover, nanocoatings can also be applied in thinner and smoother thicknesses, allowing for greater flexibility in equipment design and lower maintenance and operating costs. In this regard, organic polymeric coatings often provide corrosion protection by establishing a barrier that isolates the metal from the surrounding environment. However, all polymeric coatings are porous to damaging species such as oxygen, water, and chloride particles. This porosity accelerates the corrosion process and shortens the service life of the polymeric coating. To solve this problem, incorporating inorganic fillers into the polymeric coating formulation can reduce porosity and increase the composite coating’s lifespan. Compared with the usual filler partners, inorganic nanoparticles as fillers with very tiny grain sizes and high boundary volumes offer enhanced barrier qualities [191,192,193]. Anti-corrosion coatings create a barrier between the host material surfaces and the chemical process that occurs around them. Wear resistance and hydrophobicity are also provided by anti-corrosion nanocoatings. In this regard, anti-corrosive coatings increase the service life of metal components by up to 10 times [194]. As previously indicated, the thermal spray coating technique is one of the most modern processes. Thermal spraying, arc plasma spraying, and HVOF methods are used to commercially apply coatings. Compared with other deposition methods, the High-Velocity Flame Spray (HVFS) procedure offers a diverse approach and is a good candidate for the deposition of nanostructured composite coatings in terms of materials, deposition medium, and thickness [194,195]. Most hydro turbine components are subject to severe corrosion and erosion failure. Therefore, nickel-based coatings deposited by the HVFS process have been widely used to overcome turbine deterioration. Nickel has been selected as the candidate of matrix material because it has better fracture toughness than Al and Ti and is less expensive than Cr and Co. Corundum is referred to as alumina (Al_2_O_3_). It is a white oxide that comes in a variety of forms, including gamma, delta, theta, and alpha. High hardness, low coefficient of friction, excellent stability, high insulation, good wear qualities, and transparency are all characteristics of alumina and titania powders [196]. These are used as catalysts, insulators, surface protective coatings, and in composite materials [197,198]. Additionally, Parida et al. have stated that the mechanical properties of Ni-TiO_2_ coatings are extremely reliant on the grain boundary and particle size of the coating powders [199]. Thereupon, newer composite coatings, including nanocomposites, with a combination of higher hardness, strength, toughness, and adhesion should be studied for future applications. Eventually, this will lead to a reduction in the time and cost of corrective maintenance for hydropower plants. As regards fatigue failure, Doina et al. have concluded that the hydrodynamic loads on the blade must be reduced to decrease the peak stress value [132]. They reported that the only way to reduce stress value under current operating parameters (i.e., discharge, head, speed, and power) is to increase the number of runner blades. The fracture surfaces were exposed to chemical corrosion after the breakdown started. The presence of a high number of non-metallic inclusions enhanced and expedited this occurrence. It should be highlighted that, in today’s world, neither numerical models nor reduced-scale model testing can accurately forecast cavitation incidence in axial turbines [139]. Zhang et al. have investigated the methods of vortex identification in hydro turbines [200]. The modern solution to this phenomenon is to typically use the so-called anti-cavitation lip, which consists of a simple winglet affixed to the tip of the blades, to reduce cavitation development in axial turbines. Nevertheless, such a remedy often fails to reduce cavitation erosion, as reported by Kimon and Peter [201]. However, some researchers, based on the results of CFD analysis, have suggested creating and placing anti-cavitation lips on the runner blades as a solution to prevent tip vortex. The function of the lips was confirmed by model measurements in the hydraulic laboratory [138]. The cavitating core of the vortex was shifted further away from the suction side, and the measuring proved that the anti-cavitation lips’ installation had no effect on the turbine efficiency. Moreover, this idea was used to modify the prototype runner. After 1500 operating hours, the function of the anti-cavitation lips was examined, and no cavitation pitting or shadows on the blade or on the lips were found [138]. Figure 24 illustrates this fact.

The design of the runner blade could be altered to avoid tip vortex cavitation pitting. In this regard, changing the blade’s camber line deflection could help. Another option is to keep the tip clearance as small as possible, which is about 0.05 percent of the runner diameter. In addition, anti-cavitation lips are the third method of dealing with tip vortex cavitation. In fact, with proper design, anti-cavitation lips can significantly minimize tip vortex cavitation pitting, while having no detrimental impact on the turbine efficiency [39]. One of the clear signs of traveling bubble cavitation is the crackling noise/sound associated with cavitation, which is reported by experienced engineers. Hence, the beginning of cavitation is often detected first by this noise rather than by visual observation of the bubbles [202]. This cavity corresponds to a low incidence angle of flow, and the minimum pressure is at the impeller throat, depending on the impeller design. Accordingly, one of the effective solutions is to optimize the runner design. Early detection and treatment of cavitation phenomena through corrective maintenance is another approach for minimizing deterioration caused by traveling bubble cavitation and avoiding vibrations that can lead to significant losses of the material in hydropower plant equipment. A six-blade Kaplan turbine was studied to determine its efficiency and anticipate the cavity [203]. This study was the result of a collaboration between the University of Trista in Italy and Coléctor Turpinchetut in Slovenia. Their goal was to develop a consistent standard and to present an efficient methodology for the accurate estimation and optimization of hydro equipment performance. The numerical simulations were performed at one operating point for the maximal angle of runner blade and nominal head. The results were compared with the measured sigma break curve and the observation of cavity size on the test rig. Simulations in the steady state projected a significant but insufficient efficiency level and a modest amount of cavitation on the runner blades. The shape and size of the projected sheet cavitation in transient simulations matched the cavitation observed on the test rig. However, the anticipated efficiency was more accurate, but the value of cavitation, or Thoma number, where efficiency declined by 1%, was a little too high. The difference in results achieved using standard and calibrated Zwart model parameters is significant [203,204].

In previous studies, thermal spray coatings have been shown to act as an extraordinary barrier between the sedimentation effect and the surface of hydraulic turbines. The preference for the thermal spray technique is due to its versatile nature in the development of a variety of coatings on any surface. The enhanced performance and durability of coated steel in erosive environments have been shown in various studies [186,205,206]. HVFS is a thermal spray technique that produces a much superior coating surface, greater upright adhesion, less porosity, and reduced oxide content. Furthermore, the cost associated with this process is lower than that of other thermal spray techniques [207,208]. Several compositions of ceramic powders are produced using different methods, such as casting and crushing, sintering, and crushing, aggregation, particle cladding, and agglomeration. Because of their unique characteristics of anti-erosion properties, most of the ceramic compositions are used for the mitigation of the erosion problem. The major components used to make hard coatings are ceramics such as Al_2_O_3_, TiO_2_, and WC. Rajeev et al. have evaluated the ceramic coating compositions of Ni-20Al_2_O_3_ and Ni-10Al_2_O_3_-10TiO_2_ coated on CA6NM turbine steel using the HVFS technique [207]. They claimed that adding TiO_2_ to coatings improved binding strength and surface roughness. Grewal et al. have used the HVFS technique to investigate the effects of Ni-Al_2_O_3_ coatings on CA6NM turbine steel [208]. According to their report, adding alumina to nickel improves the mechanical quality of coating and increases its resistance to erosion. The multi-dimensional composite carbide coating has been coated on turbine steel by Liu et al. [209] and Lalit and Arora [210]. They discovered that, compared with conventional coatings, the nanocomposite coating greatly increases erosion resistance. Vibhu et al. have investigated multi-dimensional Ni-Al_2_O_3_ metal-ceramic coating compositions on CA6NM steel [211]. The scientists discovered that adding multi-dimensional alumina to the nickel matrix results in a dense coating surface that improves erosion resistance. Additionally, several scholars have conducted studies on tungsten carbide-based coatings due to their superior mechanical properties (i.e., hardness and toughness). In addition, for the alleviation of the slurry erosion problem, the use of WC-10Co-4Cr coatings has been widely suggested [211,212,213,214,215]. However, the high cost of carbide coating powders makes them unsuitable for most applications. Compared with conventional coatings, Lima et al. found that the addition of TiO_2_ nanostructured ceramic material to the Al_2_O_3_-13TiO_2_ coating composition improves fracture propagation resistance for wear applications [216]. Because of the greater toughness property produced during the application of the coating, it displayed superior wear performance.

Predicting erosion performance for coatings is more challenging than predicting erosion performance for bulk materials, where properties such as thermal expansion, bond strength, residual stresses, and thickness must be considered [217,218]. Typically, engineers and industry professionals respond to erosion problems by replacing the degraded surface with a harder one. In this regard, good coating adhesion to the substrate is required for high energy impacts. Furthermore, while coating erosion tests show steady-state erosion rates due to microchipping mechanisms, more serious damage mechanisms associated with coating spallation, such as Hertzian ring-cone cracks, stress wave propagation cracks, and lateral crack development, can occur depending on the shape of impacting particle [219]. The morphology of crystalline grains in nanodimension modifies the properties of coating materials (the nanostructured materials have crystal grains with a size smaller than 100 nm). Thermal-sprayed nanocrystalline coatings with good hardness values are found to possess better wear performance than their counterparts made from microcrystalline powders. In this regard, the HVOF technique has been used to create nanocrystalline coatings with low porosity, higher bond strength, and increased wear properties [220]. By controlling the deposition temperature, nanostructured coatings can be synthesized by vapor deposition-based methods, such as PVD or CVD. The fine particles can be prepared by grinding coarse powders to the desired particle size or by chemical synthesis of liquids and precipitation of finely dispersed solid phases with desired composition [221]. The primary use of thermal spray nanocrystalline powder coatings was to improve substrate wear resistance (i.e., sliding, abrasion, and erosion). Additionally, experiments by scholars based on the nanocomposite powders of Ti, Mo, C, NiCr, Co, Cr_2_O_3_, Cr_3_C_2_, W, titania (TiO_2_), and other materials to improve wear resistance have yielded promising results [222]. Moreover, it has also been claimed that these nanocomposite coatings can be used as heat barriers in various types of power turbines, as photo-catalytic coatings, or as electron emitters. Co-based superalloy powders, Fe-Al, WC–Co, NiCrC, and Al_2_O_3_ nanocrystalline powders have also been utilized to improve erosion properties and protect turbine blades [223].

## 5. Conclusions

Kaplan turbines are often used to optimize the production of hydraulic power. Any abnormality in the operation of the Kaplan turbine mechanism can lead to its emergency state and, ultimately, to an accident. Defects in the operation of equipment that threaten an accident or disruption of the station’s operating schedule, but can be eliminated by the personnel on duty, must be immediately eliminated in accordance with local instructions. As a result, more research into the major causes of Kaplan turbine failures could help in improving the evaluation procedures for power plants. In this critical review, the research conducted on the common phenomena that can cause damage in Kaplan turbine was discussed, including material and physical defects, deficiencies in design, deficits in manufacturing and assembly processes, corrosion failures, fatigue failure, cavitation wear, types of cavitation in hydro turbines, hydro-abrasive problems, and hydro-erosion problems, and the practical solutions are given and discussed in detail to solve the above-mentioned problems.

## Figures and Tables

**Table 1:** A brief overview of catastrophic failures in hydraulic turbines.

Hydropower Plant and Occurrence Year	Turbine Type	Description
Turbine rated power 22 MW, head 19 m, and speed 150 rpm accident in Russia, 1960.	Kaplan	They reported that due to design flaws, the turbine blades fractured, and the power plant shut down [43,44].
Turbine accident, Hydroelectric station, Zvornik, in former Yugoslavia in 1975.	Kaplan	Analysts claimed that when system unpredictability is combined with complacency or the assumption that the problem has already been solved, design implications become considerably more difficult [43].
Turbine accidents at Ozbalt hydropower plant, former Yugoslavia, 1976.	Kaplan	The main cause of failure is not printed.
Iron gate hydropower plant (Romania and Serbia), 1984.	Kaplan	Because of its high discharge, the Kaplan turbine is prone to cavitation and damage to the runner blades. This is because the water pressure around the turbine is always very low [45].
A turbine broke down in Sweden in 1986.	Kaplan	The turbine over speed signal was recorded and the emergency stop level closed the guide vanes. Therefore, emergency closure at over speed, a more severe initial condition, was enough to result in an accident [46]. In this regard, inadequate design and lack of calculation of difficult operational requirements lead to serious accidents.
Gezhouba hydropower plant (China), 1989.	Kaplan	Cavitation phenomena and high hydraulic loads caused the runner blades on the No. 5 Kaplan turbine to wear out. The root area is usually the high-stress area and it is easy for the crack to initiate [47].
Shuikou hydropower plant (China), 2006.	Kaplan	Turbine piston rod No. 3 was damaged. A crack started as a defect and continued to spread as fatigue set in as a result of cyclic loading [48].
Sayano-Shushenskaya hydropower plant (Russia), 2009.	Francis	For the safety of the hydropower plant, the speed of the synchronous generator should stay constant. This will keep the frequency almost the same. The second turbine worked in an area that is not recommended (between 200 MW and 400 MW of power with a head of water of 210 m), which caused strong dynamic loads and vibrations. The rotating speed was controlled by the speed governor. The turbine cover was unfastened because of Unit No. 2’s avalanche-like increase in vibrations. The turbine cover flew up from the stator flange because several bolts came loose due to vibrations, and some had fatigue wear of up to 80–95%. The hydraulic unit was then separated, along with its turbine wheel [49].
Haditha hydropower plant (Iraq), 2012.	Kaplan	Runner blade damages and structural cracks due to vibration and the effect of cavitation [50].
The USA turbine accident.	Kaplan	Damage to the blade and separation of the water column [46].
Ice Harbor hydropower plant, turbine No. 2.	Kaplan	Blade damage and water column separation [51,52,53,54,55,56].
Modification and extension work to increase the capacity of the Gordon M. Shrum Generating Station on the Peace River in northern British Columbia, Canada, from 261 MW to 305 MW for each unit 1 to 5 at GSM., 2009.	Kaplan	Significant losses occurred due to the disruption in the design of hydropower facilities [57].

**Table 2:** The chemical composition of SS304 and SS316 [64].

Grade	(wt%)									
	Cr	Ni	Mn	Si	Mo	C	N	S	P	Fe
SS304	18.18	8.48	1.75	0.57	0.2	0.051	0.05	0.005	0.028	Base
SS316	18	10	2	0.75	1.66	0.08	0.1	0.03	0.045	Base

**Table 3:** The mechanical properties of SS304 and SS316 [64].

Grade	Tensile Strength (M*P*a)	Hardness (Brinell)
SS304	500–700	215 max HB
SS316	400–620	149 max HB

## Data Availability

All data are reported in the article.

## Abbreviations

Mtoe	Mega tons of oil equivalent
TWh	Terawatt hour
HPP	Hydropower Plant
HSMA	High Strength Micro Alloy
HT	Heat-Treated
HTA	Hydraulic Transient Analysis
NAB alloy	Nickel Aluminum Bronze alloy
IOD	Internal Object Damage
FOD	Foreign Object Damage
HAZ	Heat-Affected Zone
PVD	Physical Vapor Deposition
CVD	Chemical Vapor Deposition
HVOF spraying	High-Velocity Oxygen Fuel spraying
HVFS	High-Velocity Flame Spray
